# The reporting and diagnosis of uterine fibroids in the UK: an observational study

**DOI:** 10.1186/s12905-016-0320-8

**Published:** 2016-07-25

**Authors:** Elisa Martín-Merino, Mari-Ann Wallander, Susan Andersson, Montse Soriano-Gabarró, Luis Alberto García Rodríguez

**Affiliations:** 1 Spanish Centre for Pharmacoepidemiologic Research (CEIFE), Madrid, Spain; 2 Department of Public Health and Caring Science, Uppsala University, Uppsala, Sweden; 3 Global Epidemiology, Bayer Pharma AG, Berlin, Germany

**Keywords:** Uterine fibroids, Incidence, Validation, Read codes, The health improvement network

## Abstract

**Background:**

Uterine fibroids (UFs) are the most common benign tumour in women, and many undergo hysterectomy or uterus-preserving procedures (UPPs) to manage their symptoms. We aimed to validate the recording of UFs in a primary care database, The Health Improvement Network (THIN), and to determine the incidence of UFs in the UK.

**Methods:**

In this observational study, women in THIN aged 15–54 years between January 2000 and December 2009 with no previous record of UFs, hysterectomy or UPPs were identified. Individuals were followed up until there was a Read code indicating UFs, they reached 55 years of age or died, or the study ended. Among those without a UF code, women were identified with a code for hysterectomy, UPPs or heavy menstrual bleeding (HMB). Anonymized patient profiles from each category were randomly selected and reviewed. Subsequently, primary care physicians were asked to complete questionnaires to verify the diagnosis for a randomly selected subgroup.

**Results:**

In total, 737,638 women were identified who met the initial inclusion criteria. The numbers of women with a code for UFs, hysterectomy, UPPs and HMB were 9380, 11,002, 3220 and 60,915, respectively; the proportions of confirmed cases of UFs were 88.8, 29.7, 57.7 and 15.9 %. The estimated number of women with UFs was 23,140 (64.0 % without a recorded UF diagnosis). The overall incidence of UFs was 5.8 per 1000 woman-years.

**Conclusions:**

UFs were confirmed in a high proportion of women with UF Read codes. However, almost two-thirds of cases were identified among women with a code for hysterectomy, UPPs or HMB. These results show that UFs are under-recorded in UK primary care, and suggest that primary care physicians tend to code the symptoms of UFs more often than the diagnosis.

**Electronic supplementary material:**

The online version of this article (doi:10.1186/s12905-016-0320-8) contains supplementary material, which is available to authorized users.

## Background

The frequency of uterine fibroids (UFs) in the general population is difficult to quantify because many women with UFs are asymptomatic [1]. Ultrasound examination of over 1000 randomly selected members of an urban health plan in the USA demonstrated that approximately half of premenopausal women with no previous diagnosis of UFs had evidence of UFs [2]. Analysis of 2500 consecutive hysteroscopies, of women aged 19–82 years revealed that almost one-quarter had UFs [3]. Similarly, examination of over 2000 consecutive pelvic ultrasound scans showed that 29.9 % of these women, aged 11–96 years, had UFs [4]. Heavy menstrual bleeding (HMB) is common in women with UFs [5], and diagnostic studies have detected UFs in approximately 30 % of women with HMB [3, 6]. Women with UFs may also suffer from bulk symptoms such as urinary incontinence/retention, bowel disturbance and pain [5]. The symptoms of UFs can have a severe impact on quality of life, and in some countries the presence of UFs is the most common indication for hysterectomy [7]. Patients who do not wish to undergo hysterectomy to treat UFs may opt for procedures that preserve uterine integrity such as myomectomy and uterine artery embolization, specifically used to treat UFs, and endometrial ablation, which is used mainly for the treatment of HMB [8].

For most patients with UFs, the point of entry into the UK healthcare system is a consultation with a primary care physician (PCP). The Health Improvement Network (THIN) is one of the largest national collections of data from primary care, containing anonymized patient information recorded by PCPs as part of routine clinical care. Symptoms, diagnoses and procedures are recorded using Read codes, and PCPs can provide additional information in free-text entries [9]. THIN also contains details of referrals to specialists, and diagnoses associated with these consultations may be entered retrospectively by PCPs, using Read codes or free-text comments. With the availability of multiple Read codes, there can be considerable variation in how PCPs record information, and previous studies have identified the need for bespoke search strategies that reflect the clinical and coding definitions in use [10]. For example, a study of the use of Read codes in diabetes mellitus management revealed that 25 different codes were reported in the 17 practices examined [11]. The aim of our study was to validate the recording of UFs in THIN, and to estimate the incidence of UFs in the UK general population.

## Methods

### Data source

THIN is a longitudinal research database containing data from over 3 million active patients currently registered with participating UK general practices [12]. The patients in THIN are representative of the UK population with respect to demographics and the prevalence of major conditions [13].

### Study design

**Fig. 1:**
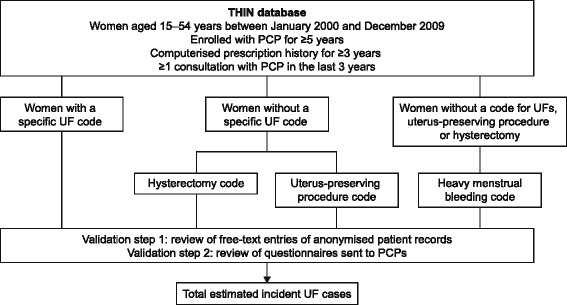
Outline of the study design. Myomectomy and uterine artery embolization are used solely for the treatment of UFs and were classified as specific UF codes. Read codes that indicated other surgeries of the uterus or endometrial ablation were classified as uterus-preserving procedures. PCP, primary care physician; THIN, The Health Improvement Network; UF, uterine fibroids

A cohort of women aged 15–54 years between January 2000 and December 2009 were identified who met the following criteria: enrolled for at least 5 years with their PCP, had a computerized prescription history for at least 3 years, and had consulted with their PCP at least once in the past 3 years (Fig. 1).

Given that myomectomy and uterine artery embolization are used solely in the treatment of UFs, women with a code for these procedures were classified as having a code for UF diagnosis. Potential UF treatments, such as endometrial ablation and uterine surgeries, were categorized under uterus-preserving procedures (UPPs). The complete list of the Read codes used can be found in Additional file 1: Table S1. The routine use of magnetic resonance image-guided focused ultrasound ablation for the treatment of UFs was not approved by the National Institute for Health and Care Excellence until 2011, so was not examined in this study [14].

Women with a Read code for UFs, hysterectomy or UPPs before the start date were excluded. Women were followed up until a Read code indicative of UFs was recorded, they reached 55 years of age or died, or the study ended. Subsequently, among women without a UF code, searches were conducted for codes for hysterectomy or UPP. Finally, a search was conducted for women with HMB who did not have a Read code for UFs, hysterectomy or UPPs.

### Validation of incident UF cases identified by a UF code

The records of a random sample of 500 women with Read codes indicative of UFs were examined, by one researcher, for free-text entries that were associated with codes for gynaecological conditions recorded at any time during the patient’s medical history. These records were also searched for comments attached to any consultation occurring within 1 month of recording the UF code. This could include additional information derived from referral letters, test results or diagnostic procedures such as ultrasound scan, hysteroscopy, laparoscopy or physical examination.

After manual review of all records, cases were assigned as probable cases (incident UF diagnosis) or non-cases (confirmed by free-text comments as negative UF diagnoses, such as adenomyosis, polyps or other leiomyoma, or prevalent cases, i.e. cases diagnosed in the past). Most probable cases of UFs were confirmed by free-text comments that referred to UF characteristics (including size, number, localization or type) or by comments related to the UFs diagnosis (e.g. confirmation by ultrasound scan or noted during pregnancy). Patients without free-text comments specifically excluding or confirming the diagnosis of UFs were retained as probable cases.

In a subsequent step, questionnaires were sent to the PCPs of 200 patients randomly sampled from this subgroup of 500 women. PCPs were requested to confirm or refute the diagnosis of UFs, and were also asked to send a copy of all UF-related information, including test results and diagnostic procedures. The proportion of women with incident UFs among those originally assigned as probable cases or non-cases was then determined.

### Validation of incident UF cases among women with a code for hysterectomy, UPPs or HMB

The profiles of a random selection of 200 women without UF codes but with codes for hysterectomy (149 records) or UPPs (51 records) were reviewed. Among those without a code for UFs, hysterectomy or UPPs, profiles of women with a code for HMB (200 records) were also reviewed. For a random sample of women from each of these three cohorts, questionnaires were sent to their PCPs requesting confirmation of whether or not the presence of UFs was the indication for the hysterectomy, UPP or HMB (or indicating the real cause otherwise). Questionnaires were sent to 50, 10 and 50 PCPs of women in the hysterectomy, UPP and HMB cohorts, respectively. For each cohort, the proportion of women with incident UFs among those originally assigned as probable cases or non-cases was determined.

### Estimation of incidence of UFs

The incidence of UFs was determined by dividing the number of women with Read codes for UFs by the total person-years contribution to first follow-up. This calculation was performed for the overall study population and for each 5-year age group of women aged 15–54 years. The corrected overall incidence of UFs included the estimated number of women with incident UFs among the cohorts with Read codes for hysterectomy, UPP or HMB, in addition to those with Read codes for UFs.

## Results

**Fig. 2:**
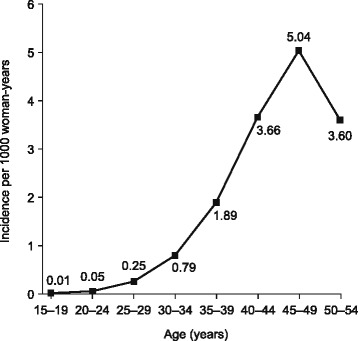
Incidence of uterine fibroids according to age group in women with a Read code for uterine fibroids

In total, 737,638 women were identified who met the initial criteria. The age distribution of the study cohort can be found in Additional file 2: Table S2. After a mean follow-up of 5.4 years, (almost 4 million person-years), 9431 women had a Read code indicating a diagnosis of UFs. Following exclusion of 51 cases in which the code was not specific to leiomyomas of the uterus, 9380 women with potential incident UFs were identified. The incidence of recorded UFs increased with age, peaking in the group aged 45–49 years (Fig. 2). Most cases of incident UFs (*n* = 7696, 82.4 %) were diagnosed in women aged 40–54 years. The numbers of women identified with a Read code for hysterectomy, UPPs and HMB were 11,002, 3220 and 60,915, respectively.

### Validation of incident UF cases identified by a UF code

In our sample of 500 patient profiles with Read codes indicative of a UFs diagnosis, 470 (94.0 %) were classified as probable incident cases of UFs following assessment of free-text comments (Table 1). The remaining 30 women (6.0 %) included 21 prevalent cases of UFs and nine women with diagnoses other than UFs. In the second validation step, of the 200 questionnaires sent, 186 valid questionnaires were received from PCPs. Of these, 176 were for patients who had been classified as probable UFs cases in the initial step; 164 patients were confirmed as having incident UFs (confirmation rate, 93.2 %). Ten valid questionnaires were returned for women who were originally classified as non-UF cases; two (20.0 %) of these were revealed as incident cases of UFs. The weighted percentages of these confirmation rates were 87.6 and 1.2 % in probable and non-UF cases, respectively.

**Table 1:** Determination of incident cases of uterine fibroids in The Health Improvement Network database

		Uterine fibroids		Hysterectomy		Uterus-preserving procedures		Heavy menstrual bleeding	
Validation step 1: review of free-text comments for verification of Read codes	Event status	Probable cases	Non-cases	Probable cases	Non-cases	Probable cases	Non-cases	Probable cases	Non-cases
	Number of records	500		149		51		200	
	Percentage	94.0 %	6.0 %	92.6 %	7.4 %	92.3 %	7.8 %	86.5 %	13.5 %
Validation step 2: results of PCP questionnaires	Questionnaires sent	200		50		10		50	
	Valid questionnaires received	176	10	34	3	8	1	38	6
	Incident cases of UFs	164 (93.2 %)	2 (20.0 %)	10 (29.4 %)	1 (33.3 %)	5 (62.5 %)	0 (0 %)	7 (18.4 %)	0 (0 %)
Weighted percentage of confirmation rates		87.6 %	1.2 %	27.2 %	2.5 %	57.7 %	0 %	15.9 %	0 %
Combined total (proportion of women correctly identified by Read codes)		88.8 %		29.7 %		57.7 %		15.9 %	
Cohort size		9380		11,002		3220		60,915	
Estimated number of incident cases in the cohort		8329		3268		1858		9685	

*PCP* primary care physician, *UFs* uterine fibroids

Taking the weighted sum of these confirmation rates, the proportion of women correctly identified by Read codes (i.e. the positive predictive value) was 88.8 %. Based on this, the total estimated number of incident cases of UFs within the cohort was 8329, with a corresponding incidence of 2.1 per 1000 woman-years.

### Validation of incident UF cases among women with a code for hysterectomy

Among the sample of 149 individuals with a Read code for hysterectomy, the index event was confirmed in 92.6 % of women by analysis of free-text comments (Table 1). From a total of 37 valid PCP questionnaires, diagnosed UFs were confirmed in 11 cases. Of these, 10 were among women originally classified as probable UFs cases. After weighting, it was estimated that 3268 women (29.7 % of women with a Read code for hysterectomy) had incident UFs.

### Validation of incident UF cases among women with a code for UPPs

From review of free-text comments in patient profiles from 51 cases of UPPs, 92.3 % of cases were confirmed as probable UPPs (Table 1). From the nine PCP questionnaires received, five cases of incident UFs were reported (one in free-text entries), all in probable cases of UFs (62.5 %). After weighting, it was estimated that 1858 women (57.7 % of women with a code for UPPs) had incident UFs.

### Validation of incident UF cases among women with a code for HMB

Of the 200 patient profiles that were manually reviewed, 173 (86.5 %) were classified as probable HMB (Table 1) on the basis of the free-text comments. Among the 44 valid PCP questionnaires received, UFs were confirmed for seven patients. All of the confirmed cases were among those originally classified as probable UFs cases (18.4 %). After weighting, UFs were estimated to be present in 9685 women (15.9 %) with HMB.

### Corrected incidence of UFs

Based on these calculations, it was estimated that there were 23,140 women with incident UFs in THIN during 2000–2009. The overall incidence of UFs was 5.8 per 1000 woman-years among women aged 15–54 years.

## Discussion

Our study showed that among women with a code indicating UFs, the proportion correctly recorded was 88.8 %, highlighting the high predictive power of Read codes in this instance. This figure compares favourably with those from other studies [15]. It was estimated, however, that only about one-third of cases were identified on the basis of a specific Read code for the diagnosis of UFs. There is considerable variation in the reliability of the recording of patient information in primary care. As might be expected, conditions with subjective diagnostic criteria, such as asthma, have a lower quality of recording than conditions with specific clinical characteristics, such as diabetes mellitus [16]. It has been recommended that in the analysis of data from primary healthcare databases, the reliability of the recording of each condition being studied should be validated [17].

Based on the total estimated number of UFs cases identified using Read codes for UFs, hysterectomy, UPP and HMB, the incidence of UFs was 5.8 per 1000 woman-years. It is worth noting that this estimate reflects the incidence of clinically relevant UFs, or those found incidentally during procedures for other reasons. Given that many women with UFs remain asymptomatic, this is likely to be an underestimate of the true incidence of the condition [18, 19].

To the best of our knowledge there have previously been no figures available for the incidence of UFs in the UK. The figure determined in our study is similar to that from an analysis of the healthcare records of women in the US armed forces, in which an incidence of 57.6 per 10,000 person-years was calculated [20]. In another US study, however, the age-standardized incidence of self-reported UFs, confirmed by examination or following hysterectomy, was 8.9 per 1000 woman-years among Caucasian women and 30.6 per 1000 woman-years among African-American women [21]. Ethnicity is not systematically recorded in THIN, but the incidence determined in our study may be lower than that obtained from self-reporting. Furthermore, between the menarche and the menopause, the incidence of UFs increases with age, and the incidence of UFs in a study population will be dependent on the age distribution of the women. Although THIN has been shown to be representative of the UK population [13], there may be small differences between the age distribution of the women eligible for this study and those in the whole UK population.

One limitation of our study is that potential unrecorded cases of UFs were sought only among women with the most common symptom of UFs, HMB. A search strategy that included women who had consulted their PCP about other gynaecological symptoms potentially related to UFs, such as dysmenorrhoea or bulk symptoms, might have identified further cases. Our study, however, already used four search strategies that included a total of 140 Read codes, and it is likely that further searches would have returned a low rate of additional confirmed cases. Another potential approach to identify women with UFs would be to search prescribing data, which are recorded in THIN using Multilex codes [22]. During the study period, however, the only medical therapies licensed for the treatment of UFs in the UK (gonadotropin-releasing hormone analogues and ulipristal acetate) were indicated for preoperative therapy, and it is likely that any women prescribed these treatments would have already been identified by the existing searches. It must also be kept in mind that the incomplete response to questionnaires sent to PCPs could have affected the confirmation rates of UF cases. If a substantial proportion of the questionnaires that were not returned related to non-cases of UFs, the true incidence may be lower than the reported estimate. However, we believe that any such effects are likely to be minor, on the basis that the response rate to PCP questionnaires was almost 90 %. Moreover, there is no reason to indicate that responders and non-responders differed in terms of the proportion of confirmed UFs cases.

There are other examples of searches using Read codes that had low sensitivities. In studies of electronic health records, fewer than two-thirds of patients with diabetes mellitus were correctly identified using the specific code ‘C10 Diabetes mellitus,’ and only 17 % of patients with active seasonal allergic rhinitis could be identified on the basis of a specific Read code [10, 11]. Furthermore, the practice of recording body mass index rather than a diagnosis of obesity resulted in only 2.1 % of patients with obesity being identified using a specific Read code [15]. These examples reflect the diversity of the recording practices used by PCPs, rather than inherent problems with Read codes. In our study, and in other cases in which a similar approach has been taken, an expanded search strategy resulted in an increase in the estimated absolute number of patients with the outcome of interest. There is still, however, potential for some underestimation of the incidence of UFs. We estimated that in THIN, there were more cases of UFs present among women with a record of HMB than among those with a UF Read code. This suggests that for women with UFs, PCPs tend to record the symptoms or the procedures undertaken rather than the diagnosis. Most UFs are detected by ultrasound examination and, in the UK, PCPs do not typically have direct access to ultrasonography. Normally, diagnoses of UFs would be communicated to PCPs by specialists, but this information might not be recorded in THIN using Read codes. These factors may contribute to the apparent practice of PCPs recording symptoms rather than the diagnosis. Recently, the Fédération Internationale de Gynécologie et d’Obstétrique developed guidelines for the nomenclature and classification of causes of abnormal uterine bleeding [23]. The ‘PALM-COEIN’ classification system, which includes a sub-classification system for UFs, was designed to assist clinicians in the evaluation of patients. The widespread adoption of this system may help the future study of UFs by encouraging PCPs to record the suspected cause of HMB. New Read codes are released periodically by the National Health Service Information Authority, and it is important that this new classification system is considered in the development of future codes.

## Conclusions

The incidence of UFs in the UK was 5.8 per 1000 woman-years, as determined by the analysis of medical records. Only about one-third of women with UFs in THIN could be identified using a Read code indicating the diagnosis of UFs. These results show that UFs are under-recorded in THIN and suggest that PCPs tend to code the symptoms of UFs more often than the diagnosis.

## Abbreviations

CEIFE, Spanish centre for pharmacoepidemiologic research ; HMB, Heavy menstrual bleeding; PCP, Primary care physician; THIN, The health improvement network; UF, Uterine fibroids; UPPs, Uterus-preserving procedures.

## Additional files

Additional file 1: Table S1. Read codes indicative of uterine fibroids. This table presents a complete list of the Read codes used to identify potential cases of uterine fibroids in THIN database. The list of Read codes includes specific codes for uterine fibroids, codes for hysterectomy and uterus preserving procedures, as well as codes for heavy menstrual bleeding. (DOCX 18 kb) Additional file 2: Table S2. Age distribution of women in the study cohort. This table presents the age distribution of the 737,638 women in the study cohort. Women are grouped into eight age categories (15–19, 20–24, 25–29, 30–34, 35–39, 40–44, 45–49 and 50–54). (DOC 28 kb)
