# Supplementary material for: The reporting and diagnosis of uterine fibroids in the UK: an observational study
Source: BMC Womens Health. 2016 Jul 25;16:45. doi: 10.1186/s12905-016-0320-8 (PMC4960833; doi:10.1186/s12905-016-0320-8)
Supplement: Additional file 1: Table S1. — Read codes indicative of uterine fibroids. This table presents a complete list of the Read codes used to identify potential cases of uterine fibroids in THIN database. The list of Read codes includes specific codes for uterine fibroids, codes for hysterectomy and uterus preserving procedures, as well as codes for heavy menstrual bleeding. (DOCX 18 kb) [file 12905_2016_320_MOESM1_ESM.docx]

**Table S1 Read codes indicative of uterine fibroids**

B78..00 Uterine leiomyoma - fibroids

B78..11 Fibroids

B780.00 Submucous uterine leiomyoma

B781.00 Intramural uterine leiomyoma

B781.11 Mural fibroids

B782.00 Subserous uterine leiomyoma

B78z.00 Uterine leiomyoma NOS

B791.00 Benign neoplasm corpus uteri NEC

B791000 Benign neoplasm of endometrium NEC

B791100 Benign neoplasm of myometrium NEC

B791200 Benign neoplasm of uterine fundus NEC

B791z00 Benign neoplasm of corpus uteri NOS

B79y.00 Benign neoplasm of other specified sites of uterus

B79z.00 Benign neoplasm of uterus NOS

BBK..00 [M]Myomatousneoplasms

BBK0.00 [M]Leiomyomatousneoplasms

BBK0000 [M]Leiomyoma NOS

BBK0011 [M]Fibroid uterus

BBK0012 [M]Fibromyoma

BBK0013 [M]Leiomyofibroma

BBK0014 [M]Myofibroma

BBK0100 [M]Intravascular leiomyomatosis

BBK0300 [M]Epithelioidleiomyoma

BBK0311 [M]Leiomyoblastoma

BBK0500 [M]Cellular leiomyoma

BBK0600 [M]Bizarre leiomyoma

BBK0z00 [M]Leiomyomatous neoplasm NOS

BBK1.00 [M]Angiomyomatousneoplasms

BBK1000 [M]Angiomyoma

BBK1011 [M]Angioleiomyoma

BBK1012 [M]Vascular leiomyoma

BBK1z00 [M]Angiomyomatous neoplasm NOS

BBK2.00 [M]Myoma and myosarcoma

BBK2000 [M]Myoma

BBK2z00 [M]Myoma or myosarcomaNOS

BBK3800 [M]Smooth muscle tumour NOS

BBL2.00 [M]Adenomyoma

L115.00 Antepartum haemorrhage with uterine leiomyoma

L115.11 Antepartum haemorrhage with fibroid

L115.12 Antepartum haemorrhage with uterine fibroid

L115000 Antepartum haemorrhage with uterine leiomyoma unspecified

L115100 Antepartum haemorrhage with uterine leiomyoma - delivered

L115200 Antepartum haemorrhage with uterine leiomyoma - not deliv

L115z00 Antepartum haemorrhage with uterine leiomyoma NOS

L241.11 Uterine fibroids in pregnancy, childbirth and the puerperium

L241000 Tumour of uterine body affecting obstetric care

L241011 Uterine fibroid affecting obstetric care

L241100 Tumour of uterine body - baby delivered

L241111 Uterine fibroid - baby delivered

L241200 Tumour of uterine body - baby delivered + p/ncomplication

L241211 Uterine fibroid - baby delivered + postpartum complication

L241300 Tumour of uterine body complicating a/n care, baby not deliv

L241311 Uterine fibroid complicating a/n care, baby not delivered

L241400 Tumour of uterine body complicp/n care, baby prevdelivered

L241411 Uterine fibroid complicating p/n care - baby delivered prev

L241z00 Uterine body tumour in pregnancy/childbirth/puerperiumNOS

L241z11 Uterine fibroid in pregnancy/childbirth/puerperiumNOS

15A9.00 H/O: myomectomy/hysterotomy

15A9.12 H/O: myomectomy

7A54A00 Percembolisation of uterine fibroid using fluoroscopic guid

7E06100 Open myomectomy

7E06111 Excision fibroid

7E06112 Myomectomy

7E06200 Open excision of lesion of uterus NEC

7E06y11 Vaginal myomectomy

7E0D012 Endoscopic myomectomy

7E0D013 Myomectomy

7E0D015 Hysteroscopicmyomectomy

7E0DC00 Transcervical resection of fibroid

**Read Codes for hysterectomy and uterus-preserving procedures**

7E04.00 Abdominal excision of uterus

7E04.11 Abdominal hysterectomy

7E04300 Total abdominal hysterectomy NEC

7E04311 Bonney abdominal hysterectomy

7E04312 Hysterectomy NEC

7E04400 Subtotal abdominal hysterectomy

7E04500 Abdominal hysterectomy and bilateral salpingoophorectomy

7E04511 Abdominal hysterectomy & bilateral salpingoophorectomy (BSO)

7E04512 TAH - total abdom hysterectomy & bilateral salpingoophorect

7E04700 Abdominal hysterectomy and right salpingoopherectomy

7E04711 Abdominal hysterectomy and left salpingoopherectomy

7E04800 Abdominal hysterectomy and left salpingoophorectomy

7E04900 TAH - Tot abdom hysterectomy and BSO - bilatsalpingophorect

7E04A00 Abdominal hysterectomy with conservation of ovaries

7E04C00 Laparoscopic hysterectomy

7E04E00 Laparoscopic subtotal hysterectomy

7E04F00 Subtotal abdominal hysterectomy with conservation of ovaries

7E04G00 Total abdominal hysterectomy with conservation of ovaries

7E04y00 Other specified abdominal excision of uterus

7E04z00 Abdominal excision of uterus NOS

7E05.00 Vaginal excision of uterus

7E05.12 Vaginal hysterectomy

7E05300 Vaginal hysterectomy NEC

7E05311 Heaney vaginal hysterectomy

7E05400 Laparoscopic vaginal hysterectomy

7E05500 Vaginal hysterectomy with conservation of ovaries

7E05y00 Other specified vaginal excision of uterus

7E05y11 Ward vaginal hysterectomy

7E05z00 Vaginal excision of uterus NOS

7E06.00 Other open operations on uterus

7E06300 Open biopsy of lesion of uterus

7E06500 Incision of uterus NEC

7E06511 HysterotomyNEC

7E06700 Endometrectomy

7E06711 Endometrial laser ablation

7E06800 Repair of uterus

7E06y00 Other specified open operation on uterus

7E06z00 Open operation on uterus NOS

7E0D000 Endoscopic excision of lesion of uterus

7E0D011 Endoscopic resection of lesion of uterus

7E0D100 Endoscopic cauterisation of lesion of uterus

7E0D200 Endoscopic cryotherapy to lesion of uterus

7E0D300 Endoscopic destruction of lesion of uterus NEC

7E0D400 Endoscopic resection of lesion of uterus

7E0D500 Trans-cervical resection endometrium

7E0D600 Endometrial ablation

7E0Dy00 Other specified therapeutic endoscopic operation on uterus

7E0Dz00 Therapeutic endoscopic operation on uterus NOS

7E0F.00 Other operations on uterus

7E0Fz00 Operation on uterus NOS

7E0G.00 Other vaginal operations on uterus

7E0G000 Vaginal excision of lesion of uterus

7E0Gy00 Other specified other vaginal operation on uterus

7E0Gz00 Other vaginal operation on uterus NOS

7E0y.00 Other specified operations on uterus

7E0z.00 Uterus operations NOS

7E04B00 Lapar total abdominal hysterectbilatsalpingo-oophorectom

7E05600 Lap assist vag hysterectomy with bilatsalpingo-oophorectomy

**Read Codes for heavy menstrual bleeding**

1573.11 H/O: heavy periods

K592.00 Excessive or frequent menstruation

K592.11 Frequent menses

K592000 Menorrhagia

K592011 Heavy periods

K592z00 Excessive or frequent menstruation NOS

1573.00 H/O: menorrhagia

K5A0.00 Premenopausal menorrhagia

K5A0.11 Climacteric menorrhagia

K5A6.00 Perimenopausalmenorrhagia

K592.00 Excessive or frequent menstruation

K592.12 Hypermenorrhoea
