# Supplementary material for: The reporting and diagnosis of uterine fibroids in the UK: an observational study
Source: BMC Womens Health. 2016 Jul 25;16:45. doi: 10.1186/s12905-016-0320-8 (PMC4960833; doi:10.1186/s12905-016-0320-8)
Supplement: Additional file 2: Table S2. — Age distribution of women in the study cohort. This table presents the age distribution of the 737,638 women in the study cohort. Women are grouped into eight age categories (15–19, 20–24, 25–29, 30–34, 35–39, 40–44, 45–49 and 50–54). (DOC 28 kb) [file 12905_2016_320_MOESM2_ESM.doc]

**Table S2 Age distribution of women in the study cohort (n = 737 638)**

| **Age (years)** | **15–19** | **20–24** | **25–29** | **30–34** | **35–39** | **40–44** | **45–49** | **50–54** |
| --- | --- | --- | --- | --- | --- | --- | --- | --- |
| n | 144 172 | 42 138 | 67 610 | 97 732 | 115 460 | 103 295 | 86 191 | 81 040 |
| % | 20% | 6% | 9% | 13% | 16% | 14% | 12% | 11% |
